# Ablação de Fibrilação Atrial de Curta Duração e Alta Potência: Preditores de Sucesso e Reincidência em Longo Prazo – Uma Análise Multivariada

**DOI:** 10.36660/abc.20230837

**Published:** 2024-11-22

**Authors:** Fabricio Vassallo, Christiano Cunha, Lucas Corsino, Eduardo Serpa, Aloyr Simões, Dalton Hespanhol, Carlos Volponi Lovatto, Dalbian Gasparini, Luiz Fernando Barbosa, Andre Schmidt

**Affiliations:** 1 Hospital Santa Rita de Cássia Vitória ES Brasil Hospital Santa Rita de Cássia, Vitória, ES – Brasil; 2 Escola Superior de Ciências Santa Casa de Misericórdia de Vitória Faculdade de Medicina Vitória ES Brasil Escola Superior de Ciências da Santa Casa de Misericórdia de Vitória Faculdade de Medicina - Arritmias Cardíacas, Vitória, ES – Brasil; 3 Universidade de São Paulo Faculdade de Medicina de Ribeirão Preto Ribeirão Preto SP Brasil Universidade de São Paulo Faculdade de Medicina de Ribeirão Preto, Ribeirão Preto, SP – Brasil

**Keywords:** Fibrilação Atrial, Reincidência, Taquicardia, Ablação por Cateter

## Abstract

**Fundamento:**

A ablação ponto a ponto com uma técnica de curta duração e alta potência (HPSD) é utilizada em todo o mundo para fibrilação atrial (FA). Poucos dados estão disponíveis com HPSD e técnica de arrasto (DT).

**Objetivo:**

Realizar uma análise multivariada dos preditores clínicos e procedimentais de sucesso e reincidência em HPSD com DT.

**Métodos:**

Foram incluídos prospectivamente 214 pacientes em primeira ablação de FA em ritmo sinusal. DT com potência de radiofrequência de 50 W e força de contato (FC) de 10–20 g e 5–10 g a uma vazão de 40 mL/min foram aplicados nas paredes anterior e posterior, respectivamente. A significância estatística foi definida como p < 0,05.

**Resultados:**

143 (66,8%) homens, FA paroxística (FAP) em 124 (57,9%), com 61,1±12,3 anos e acompanhados por 32,8±13,2 meses. Após 90 dias, FA ocorreu em 43 (20,1%) pacientes, 19 (15,3%) na FAP e 24 (26,7%) na FA persistente (FAPers). A análise multivariada indicou como preditores clínicos de reincidência: idade ≥ 65 anos (p=0,006); obesidade [índice de massa corporal > 30 (p=0,009)]; pontuação CHA_2_DS_2_VASC ≥ 3 (p=0,003); e FAPers (p=0,045). O preditor procedimental de reincidência foi um aumento da frequência cardíaca < 10% (p=0,006). Os preditores de sucesso foram aumento da frequência cardíaca ≥ 30% (p=0,04) e < 60 min no tempo de átrio esquerdo (TAE) (p=0,007).

**Conclusão:**

A ablação de FA com preditores clínicos e procedimentais DT e HPSD de reincidência foram ≥ 65 anos, obesidade, CHA_2_DS_2_VASC ≥ 3, FAPers e aumento da frequência cardíaca < 10% após a ablação. Os preditores de sucesso foram um aumento ≥ 30% na frequência cardíaca e TAE baixo (< 60 min).

## Graphical abstract

**Figura Central:**
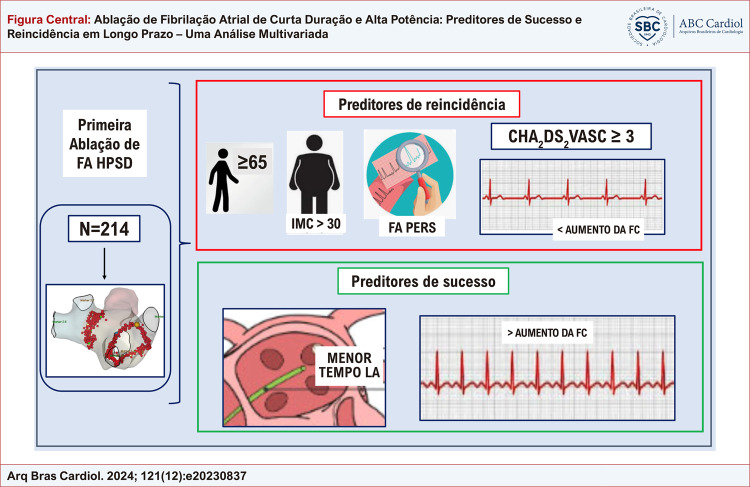
: Ablação de Fibrilação Atrial de Curta Duração e Alta Potência: Preditores de Sucesso e Reincidência em Longo Prazo – Uma Análise Multivariada

## Introdução

Vários fatores de risco estão associados ao desenvolvimento de fibrilação atrial (FA), incluindo idade, hipertensão, diabetes mellitus e insuficiência cardíaca.^1,2^ Fatores de risco menos validados incluem hipertireoidismo subclínico, obesidade e síndrome da apneia obstrutiva do sono.^1^ Os fatores de risco identificados para reincidência após ablação por cateter (AC) são menos bem estabelecidos, mas incluem o tipo de FA e parâmetros ecocardiográficos.^3,4^

Desde a publicação de Haïssaguerre et al.,^5^ que concluiu que batimentos ectópicos da veia pulmonar (VP) são gatilhos de FA, o isolamento da VP se tornou um procedimento frequente em todo o mundo. Sua principal indicação para pacientes com FA sintomática é a manutenção do ritmo sinusal na FA refratária a medicamentos como uma forma de terapia medicamentosa antiarrítmica.^6^ Altas taxas de sucesso agudo são alcançáveis, mas a eficácia a longo prazo da AC para FA continua sendo um grande desafio. Estudos anteriores documentaram que as taxas de sucesso variam de 50% a 80% dependendo do tipo de FA, sendo menores para FA persistente (FAPers).^7-10^ A maioria dos estudos utilizou uma abordagem de parâmetros de radiofrequência (RF) bem padronizados, como configurações de potência e duração e o uso de uma ablação ponto a ponto apoiada por anotações automatizadas de mapeamento eletroanatômico.

A adoção de novas técnicas e tecnologias, como a técnica de arrasto (DT) de cateter em “movimento perpétuo”^11,12^ e a nova geração de cateteres força de contato (FC)^13-16^associados a novos geradores de RF, permitiram que a técnica de alta potência e curta duração (HPSD) fosse introduzida com segurança. Identificar preditores de recorrência após a ablação de FA pode ajudar a melhorar a seleção de pacientes para esse procedimento, reduzir custos com assistência médica e evitar expor pacientes a procedimentos malsucedidos e suas complicações relacionadas.

O objetivo deste estudo foi investigar os fatores clínicos e dados de procedimentos que podem estar associados à reincidência após o tratamento da FA usando uma técnica de AC de arrasto com HPSD.

## Métodos

### Desenho do estudo

**Figura 1:**
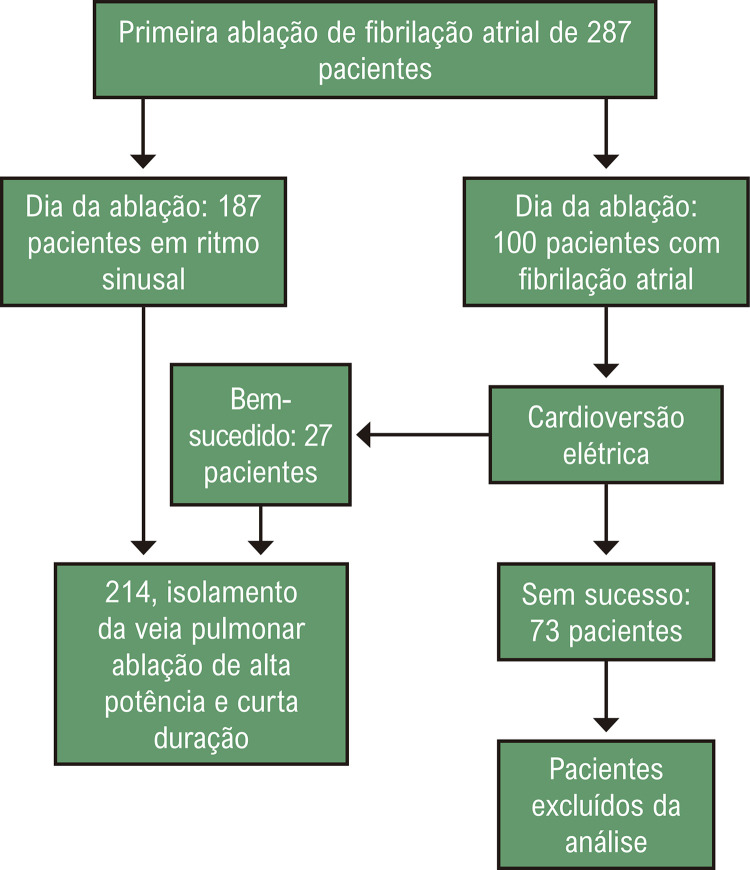
– Desenho do protocolo do estudo.

Dois centros com alto volume de ablação de FA conduziram este estudo prospectivo observacional longitudinal. Os dados foram coletados entre dezembro de 2018 e dezembro de 2021. O estudo foi aprovado pelo comitê de ética em pesquisa da instituição (CAAE 07888919.8.0000.5061). Nenhum dos pacientes havia sido submetido a procedimentos de ablação atrial esquerda e assinaram um termo de consentimento informado. Eles apresentavam FAP ou FAPers sintomática (persistente e de longa duração) que era intolerante ou refratária a pelo menos um medicamento antiarrítmico (DAA) de classe I/III. Para pacientes que não estavam em ritmo sinusal no dia do procedimento, uma cardioversão elétrica foi realizada e, se o ritmo sinusal fosse restaurado, o paciente era incluído no estudo (Figura 1).

Os pacientes foram divididos em dois grupos: aqueles com sucesso (a variável dependente – nenhum novo evento de FA) e aqueles com recorrência de FA.

### Protocolo de ablação por cateter

O protocolo é descrito em detalhes em outra sessão.^17^ Em resumo, a anticoagulação oral foi mantida e todos os pacientes foram submetidos à ablação usando um protocolo de anticoagulação oral ininterrupto. Os DAA dos pacientes foram suspensos por cinco meias-vidas antes do procedimento, exceto a amiodarona, que foi mantida apenas nos pacientes que permaneceram em FA na última consulta (30 dias) antes da ablação. Pacientes realizaram no dia ou até 48 horas antes da ablação ecocardiograma transesofágico e/ou angiotomografia do átrio esquerdo (AE) e VPs.

Foi realizado o isolamento da VP com abordagem de ablação antral utilizando o suporte do sistema de mapeamento EnSite™ Velocity™, versão 5.0, utilizando um cateter de detecção de FC, bainha defletível e sonda de ecocardiografia intracardíaca. A configuração de RF foi de 50 W com 40 mL/min^18^ de irrigação, com uma FC de 5–10 g na parede posterior e 10–20 g na parede anterior do AE, respectivamente. O cateter de ablação foi arrastado usando um movimento lento denominado “movimento perpétuo” ao redor da PV a cada 2 a 5 segundos durante o fornecimento contínuo de RF. Outras características de ablação relacionadas à FC, como o índice do tamanho da lesão (LSI) e a integral força-tempo, não foram aplicadas, pois usamos uma abordagem de movimento perpétuo ou movimento lento do cateter. Ao final do procedimento, todos os pacientes receberam uma dose de desafio de 12 mg de adenosina para cada antro da VP para desmascarar quaisquer veias dormentes. Trabalhos anteriores do nosso próprio laboratório demonstraram que essa técnica usando o AutoMarkerTM produziu um conjunto de lesões semelhante à ablação ponto a ponto.^19,20^

### Monitoramento da temperatura esofágica luminal

Em todos os pacientes, a temperatura esofágica luminal (LET) foi monitorada. O dispositivo disponível era um cateter de curvatura padrão, tamanho 7 French (7F), direcionável, simétrico, bidirecional, de curva longa, com ponta de 4 mm (EPT Blazer II, Boston Scientific, Natick, MA) com o termistor localizado na ponta. O cateter LET foi conectado diretamente ao sistema de registro de eletrofisiologia, e as mudanças de temperatura foram anotadas manualmente e exibidas no sistema de registro. Um aumento de 2 °C em relação à LET basal resultou na exibição de uma luz vermelha piscante em duas telas (para os operadores do cateter e do polígrafo). Isso forneceu um alerta para interromper imediatamente a aplicação de RF. Anteriormente, selecionar um aumento de pelo menos 1 °C levava frequentemente ao encerramento prematuro de aplicações de RF. Assim, para levar em conta as variações na LET basal, foi determinado que uma LET de 2 °C corresponde mais precisamente aos aumentos da temperatura esofágica intramural, o que pode ser prejudicial e, portanto, deve ser evitado.^21^ Geralmente, um aumento da LET de mais de 2 °C geralmente precede uma LET absoluto de 39 °C.

### Definição do efeito de isolamento de primeira passagem

O efeito de isolamento de primeira passagem (FPI) foi definido como o isolamento elétrico das veias após toda a circunferência do antro pulmonar ser circundada. Conforme definição de FPI, a estimulação foi realizada dentro e fora das veias para documentar a presença de bloqueios de saída e entrada, respectivamente.

### Definição de aumento da frequência cardíaca

Após anestesia geral e previamente à punção da veia femoral, a frequência cardíaca basal (FC) foi registrada utilizando o sistema de registro eletrofisiológico. Enquanto o procedimento estava sendo realizado e um estado de anestesia geral estava sendo mantido, a FC final também foi registrada. Qualquer diferença entre as FCs inicial e final foi calculada como uma porcentagem da mudança.

### Análise estatística

Os testes foram realizados utilizando o software Statistical Package for the Social Sciences (SPSS) versão 20 da IBM. A significância estatística foi definida como valor-p < 0,05.

Variáveis categóricas foram descritas por meio de frequências absolutas e relativas. As variáveis contínuas apresentaram distribuição normal e, portanto, foram descritas usando média ± desvio padrão. Utilizamos o teste exato de Fisher para as variáveis categóricas e o teste de Kolmogorov-Smirnov para verificar a normalidade dos dados do estudo. A comparação entre as médias dos grupos independentes foi feita com o uso do teste t não pareado.

A análise estatística utilizou a abordagem de análise de sobrevivência considerando a recorrência da FA como o evento de interesse. A vantagem desse tipo de abordagem é poder considerar a evolução do grupo de pacientes ao longo do período de observação e não apenas se houve recorrência da FA. Inicialmente, calculamos as probabilidades de sobrevivência usando o método de Kaplan-Meier. Esse tipo de análise permite avaliar apenas a influência isolada de cada variável, desconsiderando o efeito das demais. Para comparar as curvas, utilizamos o teste Log-Rank. A análise univariada foi realizada utilizando o modelo de regressão de Cox com modelo de riscos proporcionais para cada variável avaliada para estimar a razão de risco ou risco relativo.^22^ Para obter uma análise global, foi ajustada uma análise multivariada de um modelo de regressão de Cox com riscos proporcionais. Isso permite que todos os efeitos sejam avaliados ao mesmo tempo. Com esta análise multivariada, serão avaliados os fatores de risco independentemente associados à recorrência da ablação.

### Protocolo de acompanhamento pós-ablação

Os pacientes foram internados no dia do procedimento e receberam alta no dia seguinte se não tivesse ocorrido nenhuma complicação clínica ou no procedimento. Todos os pacientes foram tratados com AADs durante os primeiros 2 meses após a ablação e interrompidos depois disso, independentemente do tipo de FA. A ablação bem-sucedida foi definida como a não ocorrência de novo episódio de FA, flutter atrial e/ou taquicardia atrial com duração de pelo menos 30 segundos após um período de blanking de 3 meses. Os anticoagulantes orais foram interrompidos nos pacientes com pontuação CHA_2_DS_2_VASC menor ou igual a 3. As exceções foram pacientes que tiveram acidentes vasculares cerebrais anteriores e/ou tinham 75 anos ou mais.

Um eletrocardiograma (ECG) foi realizado após 7 dias e em 1, 2, 3, 6 e 12 meses e a cada 12 meses a partir de então. Aos 3, 6 e 12 meses e a cada 12 meses subsequentes, foi empregado o monitoramento Holter de 24 horas. Caso apresentassem sintomas de arritmia, um ECG era agendado para o mesmo dia da comunicação e um monitoramento Holter de 48 horas para o dia seguinte. Dados de FA do marcapasso foram usados quando disponíveis. Cada paciente foi ensinado a verificar o pulso manualmente ou a usar um oxímetro de pulso ou aplicativo de smartphone para monitorar sua FC e/ou ritmo cardíaco em caso de sintomas ou conforme necessário.

## Resultados

### População e características dos pacientes

Para os 214 pacientes em nossa análise primária de recorrência de FA, as características clínicas foram as seguintes: havia 143 homens (66,8%) com idade média de 61,1 ± 12,3 anos. O tempo médio de acompanhamento foi de 32,8 ± 13,2 meses. O padrão basal da FA foi paroxístico em 124 (57,9%) pacientes e persistente em 90 (42,1%). O tempo médio entre o início da FA e a inclusão no estudo foi de 11,3 ± 8,6 meses e a pontuação média CHA_2_DS_2_VASc foi de 2,4 ± 1,7.

As características clínicas que mais impactaram a recorrência foram idade ≥ 65 anos, IMC (índice de massa corporal) > 30, CHA_2_DSVASC_2_ ≥ 3, tipo FAPers e presença de apneia obstrutiva do sono. As características clínicas dos pacientes nos grupos de sucesso e recorrência são descritas em detalhes na Tabela 1.

**Tabela 1:** – Características demográficas e clínicas basais. Tempo entre ablação e reincidência

Característica clínica	Sucesso (171)	Reincidência (43)	Valor-p
Idade média (anos)	60,1 ± 12,3	65,5 ± 12,1	0,54
Homens (%)	116 (67,8)	30 (69,8)	0,12
Peso médio (kg)	81 ± 16,1	81,6 ± 14,2	0,87
Altura média (m)	1,71 ± 1,1	1,7 ± 0,8	0,91
Hipertensão (%)	116 (67,8)	28 (65,1)	0,19
Apneia obstrutiva (%)	82 (48,0)	31 (72,1)	0,003
Doença arterial (%)	55 (32,2)	18 (41,9)	0,04
Diabetes (%)	33 (19,3)	7 (16,3)	0,56
AVC (%)	11 (6,4)	4 (9,3)	0,09
Insuficiência cardíaca (%)	24 (14,0)	5 (11,7)	0,61
FA paroxística (%)	104 (60,8)	20 (46,5)	0,05
Média CHA_2_DS_2_VASC (DP)	2,23 ± 1,6	2,9 ± 1,7	0,15
Diâmetro médio do átrio esquerdo (mm)	40,6 ± 7,1	42,8 ± 8,4	0,72
Fração média de ejeção do ventrículo esquerdo (%)	61,7 ± 9,8	59,9 ± 9,9	0,81
Tempo médio (meses): diagnóstico para ablação	12,7 ± 9,0	21,46 ± 24,5	0,001
Acompanhamento médio (meses)	30,2 ± 21,2	34,2 ± 21,1	0,48

AVC: acidente vascular cerebral; FA: fibrilação atrial; DP: desvio padrão.

### Resultados do procedimento

Foram avaliados 171 (79,9%) pacientes no grupo de sucesso e 43 (20,1%) pacientes no grupo de recorrência, incluindo 19 (15,3%) do grupo FAP e 24 (26,7%) do grupo FAPers.

Os resultados da ablação dos pacientes nos grupos de sucesso e recorrência são descritos em detalhes na Tabela 2.

**Tabela 2:** A ablação resulta em casos de sucesso e reincidência

Resultados da Ablação	Sucesso (171)	Reincidência (43)	Valor-p
Tempo AE médio	57,9 ± 18,9	77,3 ± 21,2	0,001
Tempo de ablação	69,9 ± 30,2	93,1 ± 23,3	0,01
Tempo médio de RF (s)	1.478 ± 321,2	1.888,4 ± 584,1	0,001
Tempo médio de raio-X	7,6 ± 9,3	6,3 ± 5	0,1
Efeito FPI (%)	136 (77,71)	32 (82,05)	0,24
Frequência cardíaca inicial média (bpm)	53,8 ± 9,7	57,0 ± 7,7	0,28
Frequência cardíaca final média (bpm)	66,8 ± 11,6	64,4 + 10,4	0,19
Delta da frequência cardíaca média (bpm)	13 (24.16)	7,4 (12,98)	0,01
Elevação LET	58 (33,14)	21 (53,85)	0,01

AE: átrio esquerdo; RF: radiofrequência; FPI: isolamento de primeira passagem; LET: temperatura esofágica luminal.

### Análise clínica e característica da ablação

Com base nos resultados do teste log-rank, houve diferenças estatísticas indicando resultados favoráveis para os pacientes com as características acima mencionadas (< 65 anos, IMC ≤ 30, pontuação CHADSVASC < 3, FAP e sem apneia obstrutiva do sono).

Comparações de tempo até o evento de arritmias recorrentes, analisadas no período pós-blanking (com “tempo zero” ocorrendo 90 dias após o procedimento de ablação), foram realizadas entre os grupos (sucesso e recorrência) usando um modelo de regressão multivariada de Cox com riscos proporcionais.^22^ O modelo de Cox utilizou um ajuste para as seguintes covariáveis clínicas basais pré-especificadas: sexo, faixa etária no momento da inscrição (< 65 e ≥ 65 anos), presença de obesidade com IMC > 30 (presente ou ausente), tipo de FA (paroxística ou persistente), anos desde o início da FA, pontuação CHA_2_DS_2_-VASc (0–2 ou ≥ 3), hipertensão (presente ou ausente), acidente vascular cerebral isquêmico (presente ou ausente), diabetes (presente ou ausente), insuficiência cardíaca clínica (presente ou ausente), doença arterial ou coronária (presente ou ausente), insuficiência renal crônica com depuração de creatinina < 30 mL/min (presente ou ausente), apneia obstrutiva do sono com apneia moderada ou grave (presente ou ausente), aumento do AE (diâmetro do AE ≤ 42 ou > 42 mm) e fração de ejeção (< 50 ou ≥ 50%). A mesma análise foi feita usando características intraprocedimentais: elevação da frequência cardíaca < 10% do nível basal (sim vs. não), elevação da frequência cardíaca ≥ 30% (sim ou não), TAE < 60 min (sim vs. não), tempo total do procedimento < 80 min (sim vs. não), tempo de RF < 1.500 s (sim vs. não), efeito de isolamento de primeira passagem (sim vs. não) e elevação da LET (sim vs. não).

**Figura 2:**
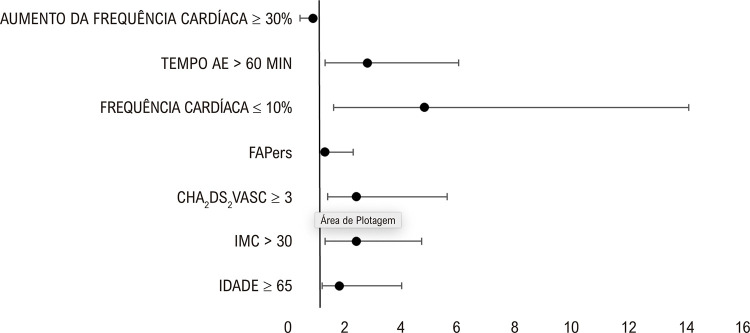
– Gráfico em floresta mostrando os 7 preditores de sucesso/reincidência (razão de risco com IC de 95%). IMC: índice de massa corporal; AE: átrio esquerdo; FAPers: fibrilação atrial persistente.

Usando análise multivariada, os preditores de recorrência foram idade > 65 anos; obesidade/IMC > 30; CHA_2_DS_2_VASC ≥ 3; tipo de FA, com FAPers apresentando maior taxa de recorrência; aumento da frequência cardíaca ≤ 10%. Encontramos dois preditores de sucesso: TAE < 60 min e um aumento da frequência cardíaca após ablação de ≥ 30 (Tabela 3 e Figura 2).

**Tabela 3:** – Análise multivariada das características clínicas e resultados da ablação que impactam o sucesso e a reincidência

Preditores	Sucesso	Reincidência	Valor-p
Idade (anos)	< 65	≥ 65	0,006
Obesidade (IMC)	≤ 30	> 30	0,009
CHA_2_DS_2_VASC	≤ 2	≥ 3	0,003
FA persistente	NÃO	SIM	0,045
Aumento de FC ≤ 10%	NÃO	SIM	0,006
Tempo AE < 60 min	SIM	NÃO	0,007
Aumento da FC ≥ 30%	SIM	NÃO	0,04

IMC: índice de massa corporal; FA: fibrilação atrial; AE: átrio esquerdo; FC: força de contato.

Durante o estudo, tivemos uma complicação importante, derrame pericárdico, que exigiu drenagem percutânea do pericárdio. Dois pseudoaneurismas também foram observados no estudo com injeção de trombina e resolução.

## Discussão

Vários estudos sobre FA examinaram os resultados da ablação usando cateteres de detecção de FC e HPSD, incorporando características clínicas. Nosso foco foi diferente dos estudos anteriores, que destacaram principalmente os fatores clínicos tradicionais como preditivos de resultados piores. A maioria deles não analisou a associação entre essas características clínicas e os resultados do procedimento especificamente com o uso de HPSD associado à DT de cateter em “movimento perpétuo”.

Um estudo anterior^21^ mostrou os seguintes seis preditores independentes de recorrência de FA após ablação inicial de FC em pacientes submetidos à ablação por HPSD: idade avançada, sexo feminino, FAPers e de longa duração vs. paroxística, tamanho maior do AE, isolamento da parede posterior e uso de SmartTouch vs. TactiCath. PWI foi analisado e associado a piores resultados para todos os tipos de FA, exceto FA paroxística, e o único preditor de recorrência relacionada ao procedimento foi o tipo de cateter (marca).^23^

Assim como nos resultados dos recentes estudos EARLY-AF^24^ e PROGRESSIVE-AF,^25^ o tempo entre o diagnóstico e a AC teve um grande impacto na taxa de sucesso em nosso estudo, demonstrado pelos tipos de FA. Nesses estudos, uma menor taxa de recorrência foi observada em pacientes designados para crioablação do que naqueles que receberam DAA. No estudo EARLY-AF,^24^ as taxas de recorrência nos grupos de ablação e medicação foram de 42,9% e 67,8%, respectivamente. Em um cenário de acompanhamento extenso, a presença de FAPers foi menor no grupo de ablação em comparação ao grupo de medicação. Taquiarritmias atriais foram detectadas em 56,5% e 77,2% dos grupos de ablação e medicação, respectivamente. Neste manuscrito, descrevemos uma maior incidência de recorrência no grupo FAPers, de 26,7%, em comparação ao grupo FAP, com 15,3%. Nosso tempo entre o diagnóstico de FA e a ablação nos grupos de sucesso e recorrência foi de 12,7 ± 9,0 e 21,46 ± 24,5 meses (p=0,01), respectivamente, mostrando que atrasos no diagnóstico e encaminhamento para ablação também impactam os resultados em longo prazo.

Outro achado interessante e importante é o impacto do IMC nos resultados da ablação. Maior taxa de recorrência foi demonstrada em pacientes com IMC > 30. Este é um preditor de maiores taxas de recorrência, como um estudo observacional europeu^26^ que mostrou uma recidiva de arritmia atrial em 12 meses em 43,6% em pacientes obesos e 48% em pacientes obesos mórbidos.

Em um registro alemão,^27^ os preditores de recorrência clinicamente observados foram gênero feminino e maior probabilidade de FAPers de longa duração. Comorbidades como insuficiência renal e doença valvar cardíaca foram significativamente mais frequentes em pacientes com recorrência. Além disso, pacientes com recorrência tiveram maior probabilidade de apresentar-se na classe NYHA ≥ II. Menor recorrência foi observada em pacientes com FAP. As características procedimentais relacionadas à reincidência foram menor energia durante a aplicação de RF, menor tempo de RF, maiores doses de fluoroscopia e recidiva hospitalar após a ablação. Analisando todas as características clínicas mencionadas acima, a pontuação CHA_2_DAS_2_VASC resumiu o impacto da comorbidade como um marcador de maior taxa de recorrência. Neste manuscrito, uma pontuação CHA_2_DAS_2_VASC ≥ 3 indicou um aumento de 2,4 vezes na recorrência.

Em contraste com os resultados do nosso estudo, outro estudo^28^ indicou que a taxa de recorrência livre de dois anos foi significativamente melhor no grupo FPI do que no grupo não FPI. Tanto no grupo de sucesso quanto no de recorrência do presente estudo, observamos uma alta taxa de FPI e a recorrência desses pacientes pode estar associada a gatilhos não venosos pulmonares.

Enquanto isso, outra equipe de pesquisa^29^ descobriu que a interrupção abrupta da RF devido a alertas de temperatura esofágica afetou a reconexão aguda e crônica da VP. Eles também descobriram que as veias que causavam alertas de temperatura e interrompiam aplicações de energia de RF não tinham mais probabilidade de se reconectar de forma aguda ou crônica do que as veias que tinham lesões de RF em dose total sem causar alertas. Em um estudo randomizado subsequente, o mesmo grupo^30^ descobriu que na AC guiada por LSI, o uso de maior potência não resultou em um número maior de alertas ou picos de temperatura esofágica do que a menor potência. De fato, isso poderia ter levado a um número menor. Alta potência pareceu estar associada a melhores resultados em procedimentos agudos; além disso, a ausência de um maior número de alertas esofágicos com o uso de maior potência pode estar associada à obtenção de melhores lesões de RF.

Um estudo por Yu et al.^31^ revelou que a modificação do ritmo sinusal da FC foi indicativa de alta manutenção do ritmo sinusal pós-ablação de FA com modulação vagal significativa e sem efeitos cardíacos adversos. Nesses casos de ablação pós-FA, uma alta FC sinusal foi associada a uma recorrência clínica significativamente menor de FA após AC. Esses achados foram corroborados por von Olshausen et al.^32^ que mostraram um aumento significativo de 11,5 bpm imediatamente após a ablação. Três meses após a ablação, a FC média diminuiu ligeiramente, porém permaneceu bem mais alta que o valor pré-ablação. Foi sugerido que um aumento no ritmo sinusal da FC estava relacionado a uma menor probabilidade de recorrência. Corroborando os achados do manuscrito real, nosso estudo anterior^33^ usando ablação de baixa potência e longa duração (LPLD) observou recorrência em 40,4%, enquanto no grupo HPSD, a recorrência foi observada em apenas 16,5% dos pacientes ao longo de 30 meses de acompanhamento. Comparando as técnicas LPLD e HPSD, a última produziu um aumento maior da FC, com um impacto importante na ausência de recorrência. Quando reunimos os pacientes que atingiram FCs mais altas após a ablação, independentemente da técnica utilizada, o aumento da FC também mostrou um impacto importante na ausência de recorrência. Outra questão importante abordada no presente estudo foi a documentação de duas variáveis na modulação da FC: a incapacidade de atingir um aumento de > 10% na FC com risco de recorrência de 4,8 vezes e a presença de um aumento de ≥ 30% na FC com chance 1,2 vez maior de manutenção do ritmo sinusal durante nosso acompanhamento.

Até onde sabemos, este é o primeiro estudo a documentar que o tempo de AE inferior foi um marcador importante de sucesso a longo prazo, com um risco 2,8 vezes menor de recorrência. Isso pode ser um reflexo de um melhor preenchimento do conjunto de lesões, já que as VPs foram isoladas de forma mais rápida.

Como questão de prognóstico, os achados do nosso estudo podem ser úteis na seleção de pacientes que precisarão de um acompanhamento mais próximo com um protocolo de monitoramento mais rigoroso, bem como durante a AC, uma vez que características intraprocedimentais podem identificar pacientes de maior risco para reincidência de FA. Em relação à modulação da FC, ela também pode ser útil para indicar uma possível modalidade de tratamento em pacientes que precisam de ablação repetida e não apresentam alteração significativa na FC.

### Limitações do estudo

Neste estudo piloto prospectivo, de braço único e centro único, o ritmo cardíaco não contínuo foi avaliado usando ECGs e monitoramento Holter de 24 horas para documentar a recorrência de taquiarritmia atrial. O monitoramento contínuo pode produzir achados diferentes, mas esse tipo de recurso não estava disponível rotineiramente em nossas instituições e os pacientes sintomáticos receberam um monitoramento mais contínuo, sem impacto na documentação de arritmias atriais.

Outra limitação do nosso estudo foi a exclusão dos pacientes que não converteram para ritmo sinusal após cardioversão elétrica. Esses pacientes têm um tipo estabelecido de FA com maiores taxas de recorrência que não foi analisado no estudo atual, e isso pode ser um viés de seleção. O principal motivo para a seleção de pacientes apenas em ritmo sinusal no início do procedimento, conforme descrito anteriormente, foi a necessidade de analisar a modificação da frequência cardíaca sinusal após a ablação. Por fim, devemos ressaltar que quando incluímos apenas pacientes em ritmo sinusal no início da ablação pode haver também outra forma de viés de seleção.

## Conclusões

Neste estudo, usando HPSD e DT, identificamos dois preditores de sucesso: um aumento de ≥ 30% na frequência cardíaca e menor TAE (< 60 min). As características do procedimento clínico de maior risco que indicaram recorrência foram idade ≥ 65 anos, obesidade, pontuação CHA_2_DS_2_VASC ≥ 3, FAPers e aumento da frequência cardíaca de ≤ 10% após a ablação.
