# Supplementary material for: Ablação de Fibrilação Atrial de Curta Duração e Alta Potência: Preditores de Sucesso e Reincidência em Longo Prazo – Uma Análise Multivariada
Source: Arq Bras Cardiol. 2024 Nov 22;121(12):e20230837. [Article in Portuguese] doi: 10.36660/abc.20230837 (PMC11634315; doi:10.36660/abc.20230837)
Supplement: Translation

# Translation: High Power Short Duration Atrial Fibrillation Ablation: Long-Term Predictors of Success and Recurrence – A Multivariate Analysis

**DOI:** 10.36660/abc.20230837i

**Authors:** Fabricio Vassallo, Christiano Cunha, Lucas Corsino, Eduardo Serpa, Aloyr Simões, Dalton Hespanhol, Carlos Volponi Lovatto, Dalbian Gasparini, Luiz Fernando Barbosa, Andre Schmidt

**Affiliations:** 1 Brazil Hospital Santa Rita de Cássia, Vitória, ES – Brazil; 2 Brazil Escola Superior de Ciências da Santa Casa de Misericórdia de Vitória Faculdade de Medicina - Arritmias Cardíacas, Vitória, ES – Brazil; 3 Brazil Universidade de São Paulo Faculdade de Medicina de Ribeirão Preto, Ribeirão Preto, SP – Brazil

**Keywords:** Atrial Fibrillation, Recidivism, Tachycardia, Catheter Ablation

## Abstract

**Background:**

Point-by-point ablation with a high-power short-duration (HPSD) technique in atrial fibrillation (AF) ablation is used worldwide. Little data is available with the HPSD and dragging technique (DT).

**Objective:**

To perform a multivariate analysis of clinical and procedural predictors of success and recurrence in HPSD with DT.

**Methods:**

214 patients in the first AF ablation in sinus rhythm were prospectively enrolled. DT with radiofrequency power of 50 W and contact force (CF) of 10–20 g and 5–10 g at a flow rate of 40 mL/min were applied on the anterior and posterior walls, respectively. Statistical significance was defined as p < 0.05.

**Results:**

143 (66.8%) males, paroxysmal AF (PAF) in 124 (57.9%), with 61.1±12.3 years and followed for 32.8±13.2 months. After 90 days, AF occurred in 43 (20.1%) patients, 19 (15.3%) from PAF, and 24 (26.7%) in persistent AF (PersAF). Multivariate analysis indicated as clinical predictors of recurrence: age ≥ 65 years (p=0.006); obesity [body mass index > 30 (p=0.009)]; CHA_2_DS_2_VASC score ≥ 3 (p=0.003); and PersAF (p=0.045). The procedural predictor of recurrence was a heart rate increase < 10% (p=0.006). Predictors of success were an increase in heart rate ≥ 30% (p=0.04) and < 60 min in left atrium time (LAT) (p=0.007).

**Conclusion:**

AF ablation with DT and HPSD clinical and procedural predictors of recurrence were ≥ 65 years, obesity, a CHA_2_DS_2_VASC ≥ 3, PersAF, and a heart rate increase of < 10% after ablation. Success predictors were an increase of ≥ 30% in heart rate and low LAT (< 60 min).

## Introduction

Numerous risk factors are associated with the development of atrial fibrillation (AF), including age, hypertension, diabetes mellitus and heart failure.^1,2^ Less validated risk factors include subclinical hyperthyroidism, obesity, and obstructive sleep apnea syndrome.^1^ Identified risk factors for recurrence after catheter ablation (CA) are less well established but include the type of AF and echocardiographic parameters.^3,4^

Since the publication of Haïssaguerre et al.,^5^ which concluded that ectopic beats from a pulmonary vein (PV) are AF triggers, PV isolation has become a frequent procedure worldwide. Its major indication for symptomatic AF patients is the maintenance of sinus rhythm in drug-refractory AF as a form of anti-arrhythmic drug therapy.^6^ High acute success rates are achievable, but long-term efficacy of CA for AF remains a major challenge. Previous studies have documented that success rates vary from 50% to 80% depending on the type of AF, being lower for persistent AF (PersAF).^7-10^ Most studies used well-standardized radiofrequency (RF) parameters approach such as power and duration settings and the use of a point-by-point ablation supported by electroanatomic mapping automated annotations.

The adoption of new techniques and technology, such as a catheter dragging “perpetual motion” technique^11,12^ and a new generation of contact force (CF) catheters^13-16^associated with new RF generators, permitted the high-power short-duration (HPSD) technique to be safely introduced. Identifying predictors of recurrence after AF ablation may help improve patient selection for this procedure, reduce healthcare costs, and avoid exposing patients to unsuccessful procedures and their related complications.

The objective of this study was to investigate the clinical factors and procedural data that may be associated with recurrence after AF treatment using a dragging CA technique with HPSD.

## Methods

### Study design

Two centers with high volumes of AF ablation conducted this longitudinal, observational prospective study. Data were collected between December 2018 and December 2021. The study was approved by the institution’s research ethics committee (CAAE 07888919.8.0000.5061). All patients were naive to left atrial ablation procedures and signed an informed consent form. They presented symptomatic PAF or PersAF (both persistent and long-persistent) that was intolerant of or refractory to at least one class I/III antiarrhythmic drug (AAD). Electrical cardioversion was performed for patients who were not in sinus rhythm on the day of the procedure, and if sinus rhythm was restored, the patient was included in the study (Figure 1).

**Figure 1:** – Study protocol design.

The patients were divided into two groups: those with success (the dependent variable – no new AF event) and those with recurrence of AF.

### Catheter ablation protocol

The protocol is described in detail elsewhere.^17^ In summary, oral anticoagulation was maintained, and all patients underwent ablation using an uninterrupted oral anticoagulation protocol. The patients’ AADs were suspended for five half-lives prior to the procedure, except for amiodarone, which was maintained only in patients that remained in AF in the last appointment (30 days) prior to the ablation. Patients performed on the day of or up to 48 hours before ablation transesophageal echocardiography and/or angiotomography of the left atrium (LA) and PVs.

The PV isolation with an antral ablation approach using the support of the EnSite™ Velocity™ mapping system, version 5.0, using a CF sensing catheter, deflectable sheath, and intracardiac echocardiography probe was performed. The RF setting was 50 W with 40 mL/min^18^ of irrigation, with a CF of 5–10 g on the posterior and 10–20 g on the anterior LA walls, respectively. The ablation catheter was dragged using a “perpetual motion,” a slow movement around the PV every 2 to 5 seconds during continuous RF delivery. Other CF-related ablation characteristics, such as the lesion size index (LSI) and force-time integral (FTI), were not applied since we used a perpetual motion or slow catheter movement approach. At the end of the procedure, all patients were given a challenge dose of 12 mg of adenosine for each PV antrum to unmask any dormant veins. Previous work from our own laboratory demonstrated that this technique using the AutoMarker^TM^ produced a similar lesion set as point-by-point ablation.^19,20^

### Luminal esophageal temperature monitoring

In all patients, luminal esophageal temperature (LET) was monitored. The available device was a standard-curve, 7 French size (7F), steerable, symmetrical, bidirectional, long-curve, 4-mm tip catheter (EPT Blazer II, Boston Scientific, Natick, MA) with the thermistor located at the tip. The LET catheter was plugged directly into the electrophysiology recording system, and temperature changes were manually annotated and displayed on the recording system. A 2 °C increase from the baseline LET resulted in a flashing red light being displayed on two screens (for the catheter and polygraph operators). This provided an alert to stop the RF application immediately. Previously, selecting an increase of at least 1 °C led to the frequent premature termination of RF applications. Thus, to account for variations in the baseline LET, an LET of 2 °C was determined to correspond to the intramural esophageal temperature increases more accurately, which can be detrimental and should, therefore, be avoided.^21^ Generally, a LET increase of more than 2 °C usually precedes an absolute LET of 39 °C.

### First-pass isolation effect definition

The first-pass isolation (FPI) effect has been defined as the electrical isolation of the veins after the entire pulmonary antrum circumference is encircled. As per the definition of FPI, stimulation was performed inside and outside the veins to document the presence of exit and entrance blocks, respectively.

### Heart rate increase definition

After general anesthesia and prior to femoral vein puncture, basal heart rate (HR) was recorded using the electrophysiology recording system. While the procedure was being completed and a general anesthesia state was being maintained, the final HR was also recorded. Any difference between the initial and final HRs was calculated as a percentage of the change.

### Statistical Analysis

Tests were performed using IBM’s Statistical Package for the Social Sciences (SPSS) software version 20. Statistical significance was defined as p-value < 0.05.

Categorical variables were described through absolute and relative frequencies. Continuous variables presented a normal distribution and were therefore described using mean ± standard deviation. We used the Fisher’s exact test for the categorical variables and the Kolmogorov-Smirnov test was used to verify the normality of the data in the study. The comparison between the mean of the independent groups was made with the use of an unpaired t-test.

Statistical analysis used the survival analysis approach considering the recurrence of AF as the event of interest. The advantage of this type of approach is to be able to consider the evolution of the group of patients throughout the observation period and not just whether there was a recurrence of AF. Initially, we calculate surviving probabilities using the Kaplan–Meier method. This type of analysis allows evaluating only the isolated influence of each variable, disregarding the effect of the others. To compare the curves, we used the Log-Rank test. Univariate analysis was performed using the Cox regression model with a proportional risks model for each variable evaluated to estimate the hazard ratio or relative risk.^22^ To obtain a global analysis, a multivariate analysis, a Cox regression model with proportional hazards was adjusted. This allows all effects to be evaluated at the same time. With this multivariate analysis risk factors independently associated with ablation recurrence will be assessed.

### Post-ablation follow-up protocol

Patients were admitted on the day of the procedure and discharged the day after the procedure if no clinical or procedural complication occurred. All patients were treated with AADs during the first 2-month after ablation and interrupted after despite the type of AF. Successful ablation was defined as the non-occurrence of new AF, atrial flutter and/or atrial tachycardia episode lasting at least 30 sec after a 3-month blanking. Oral anticoagulants were interrupted for patients with CHA_2_DS_2_VASC scores less than or equal to 3. The exceptions were patients who had previous strokes and/or were aged 75 years or older.

An electrocardiogram (ECG) was traced after 7 days and at 1, 2, 3, 6, and 12 months and every 12 months thereafter. At 3, 6, and 12 months and every subsequent 12 months, 24-hour Holter monitoring was employed. If they experienced arrhythmia symptoms, an ECG was scheduled for the same day of the communication and a 48-hour Holter monitoring for the next day. Pacemaker AF data were used when available. Each patient was taught to check their pulse manually or to use a pulse oximeter or smartphone application to monitor their HR and/or heart rhythm in case of symptoms or as needed.

## Results

### Patient population and characteristics

For the 214 patients in our primary analysis of AF recurrence, the clinical characteristics were as follows: there were 143 males (66.8%) with a mean age of 61.1 ± 12.3 years. The mean follow-up time was 32.8 ± 13.2 months. The baseline pattern of AF was paroxysmal in 124 (57.9%) patients and persistent in 90 (42.1%). The mean time from AF onset to enrollment in the study was 11.3 ± 8.6 months, and the mean CHA_2_DS_2_VASc score was 2.4 ± 1.7.

The clinical features that most impacted recurrence were age ≥ 65 years old, BMI > 30, CHA_2_DSVASC_2_ ≥ 3, PersAF type, and the presence of obstructive sleep apnea. The clinical characteristics of the patients in the success and recurrence groups are described in detail in Table 1.

**Table 1:** – Baseline demographic and clinical characteristics. Time from ablation to recurrence

Clinical Feature	Success (171)	Recurrence (43)	p-Value
Mean Age (years)	60.1 ± 12.3	65.5 ± 12.1	0.54
Males (%)	116 (67.8)	30 (69.8)	0.12
Mean Weight (kgs)	81 ± 16.1	81.6 ± 14.2	0.87
Mean Height (m)	1.71 ± 1.1	1.7 ± 0.8	0.91
Hypertension (%)	116 (67.8)	28 (65.1)	0.19
Obstructive Apnea (%)	82 (48.0)	31 (72.1)	0.003
Arterial Disease (%)	55 (32.2)	18 (41.9)	0.04
Diabetes (%)	33 (19.3)	7 (16.3)	0.56
Stroke (%)	11 (6.4)	4 (9.3)	0.09
Heart Failure (%)	24 (14.0)	5 (11.7)	0.61
Paroxysmal AF (%)	104 (60.8)	20 (46.5)	0.05
Mean CHA_2_DS_2_VASC (SD)	2.23 ± 1.6	2.9 ± 1.7	0.15
Mean Left Atrium Diameter (mm)	40.6 ± 7.1	42.8 ± 8.4	0.72
Mean Left Ventricular Ejection Fraction (%)	61.7 ± 9.8	59.9 ± 9.9	0.81
Mean Time (months): Diagnosis to Ablation	12.7 ± 9.0	21.46 ± 24.5	0.001
Medium Follow-Up (months)	30.2 ± 21.2	34.2 ± 21.1	0.48

AF: atrial fibrillation; SD: standard deviation.

### Procedure results

There were 171 (79.9%) patients evaluated in the success group and 43 (20.1%) patients in the recurrence group, including 19 (15.3%) from the PAF and 24 (26.7%) from the persAF groups.

The ablation results of the patients in success and recurrence groups are described in detail in Table 2.

**Table 2:** – Ablation results in cases of success and recurrence

Ablation Results	Success (171)	Recurrence (43)	p-Value
Mean LA time	57.9 ± 18.9	77.3 ± 21.2	0.001
Ablation time	69.9 ± 30.2	93.1 ± 23.3	0.01
Mean RF time (sec)	1,478 ± 321.2	1,888.4 ± 584.1	0.001
Mean X-Ray time	7.6 ± 9.3	6.3 ± 5	0.1
FPI effect (%)	136 (77.71)	32 (82.05)	0.24
Mean Initial Heart Rate (bpm)	53.8 ± 9.7	57.0 ± 7.7	0.28
Mean Final Heart Rate (bpm)	66.8 ± 11.6	64.4 + 10.4	0.19
Mean Heart Rate delta (bpm)	13 (24.16)	7.4 (12.98)	0.01
LET elevation	58 (33.14)	21 (53.85)	0.01

LA: left atrium; RF: radiofrequency; FPI: first-pass isolation; LET: luminal esophageal temperature.

### Clinical and ablation characteristic analysis

Based on the results of the log-rank test, statistical differences indicated favorable outcomes for the patients with the abovementioned features (< 65 years old, BMI ≤ 30, CHADSVASC score < 3, PAF, and no obstructive sleep apnea).

Time-to-event comparisons of recurrent arrhythmias, analyzed in the post-blanking period (with “time zero” occurring 90 days after the ablation procedure), were performed between groups (success and recurrence) using a multivariate Cox regression model with proportional risks.^22^ The Cox model utilized an adjustment for the following pre-specified baseline clinical covariates: sex, age group at enrollment (< 65 and ≥ 65 years old), presence of obesity with BMI > 30 (present or absent), AF type (paroxysmal or persistent), years since onset of AF, CHA_2_DS_2_-VASc score (0–2 or ≥ 3), hypertension (present or absent), ischemic stroke (present or absent), diabetes (present or absent), clinical heart failure (present or absent), arterial or coronary disease (present or absent), chronic renal failure with creatinine clearance < 30 mL/min (present or absent), obstructive sleep apnea with moderate or severe apnea (present or absent), left atrial enlargement (LA diameter ≤ 42 or > 42 mm), and ejection fraction (< 50 or ≥ 50%). The same analysis was made using intraprocedural features: heart rate elevation of < 10% from basal (yes vs. no), heart rate elevation ≥ 30% (yes or no), LA time < 60 min (yes vs. no), total procedure time < 80 min (yes vs. no), RF time < 1,500 sec (yes vs. no), first pass isolation effect (yes vs. no), and luminal esophageal temperature (LET) elevation (yes vs. no) (Table 4).

Using multivariate analysis, the predictors of recurrence were age > 65 years; obesity/BMI > 30; CHA_2_DS_2_VASC ≥ 3; AF type, with PersAF showing a higher recurrence rate; heart rate increase ≤ 10%. We found two predictors of success: an LA time of < 60 min and a heart rate increase after ablation of ≥ 30 (Table 3 and Figure 2).

**Table 3:** – Multivariate analysis of clinical features and ablation results impacting success and recurrence

Predictors	Success	Recurrence	p-Value
Age (years)	< 65	≥ 65	0.006
Obesity (BMI)	≤ 30	> 30	0.009
CHA_2_DS_2_VASC	≤ 2	≥ 3	0.003
Persistent AF	NO	YES	0.045
HR increase ≤ 10%	NO	YES	0.006
LA Time < 60 min	YES	NO	0.007
HR increase ≥ 30%	YES	NO	0.04

AF: atrial fibrillation; BMI: body mass index; LA: left atrium.

**Figure 2:** – Forest plot graphic showing the 7 predictors of success/recurrence (hazard ratio with 95% CI). HR: heart rate; LA: left atrium; PersAF: persistent atrial fibrillation; BMI: body mass index.

During the study, we had one major complication, a pericardial effusion, which required percutaneous pericardial drainage. Two pseudoaneurysms were also observed in the study with thrombin injection and resolution.

## Discussion

Several AF studies have examined the outcomes of ablation using CF-sensing catheters and HPSD while incorporating clinical characteristics. Our focus was different from those of previous studies, which highlighted mostly the traditional clinical factors as a predictive of poorer outcomes. Most of them did not analyze the association between these clinical characteristics and procedural results specifically with the use of the HPSD associated with a “perpetual motion” catheter dragging technique (DT).

Previous study^21^ showed the following six independent predictors of AF recurrence after initial CF ablation in HPSD ablation patients: older age, female gender, persistent and longstanding vs. paroxysmal AF (PAF), larger LA size, posterior wall isolation, and use of SmartTouch vs. TactiCath. PWI was analyzed and associated with worse outcomes for all types of AF except PAF, and the only predictor of procedure-related recurrence was the catheter type (brand).^23^

As in the results of the recent EARLY-AF^24^ and PROGRESSIVE-AF^25^ trials, the time from diagnosis to CA had a major impact on the success rate in our study demonstrated by the types of AF. In those studies, a lower recurrence rate was observed in patients assigned to cryoablation than in those who received AAD. In the EARLY-AF^24^ trial, the recurrence rates in the ablation and medication groups were 42.9% and 67.8%, respectively. In a long follow-up setting, the presence of PersAF is lower in the ablation compared to the medication group. Atrial tachyarrhythmias were detected in 56.5% and 77.2% of the ablation and medication groups, respectively. In this manuscript, we described a higher incidence of recurrence in the PersAF group, at 26.7% compared to the PAF group with 15.3%. Our time from AF diagnosis to ablation in the success and recurrence groups were 12.7 ± 9.0 and 21.46 ± 24.5 months (p=0.01), respectively, showing that delays in diagnosis and referral to ablation also impact long-term results.

Another interesting and important finding is the impact of BMI on the ablation results. A higher recurrence rate was shown in patients with a BMI > 30. This is a predictor of higher recurrence rates as a European observation study^26^showed an atrial arrhythmia relapse at 12 months in 43.6% of obese and 48% of morbidly obese patients.

In a German registry,^27^ the clinically observed recurrence predictors were female gender and higher likeliness of longstanding persAF. Comorbidities, such as renal failure and valvular heart disease, were significantly more frequent in patients with recurrence. In addition, patients with recurrence were more likely to present in NYHA class ≥ II. Lower recurrence was observed in patients with PAF. Procedural features related to recurrence were lower energy during RF application, lower RF time, higher doses of fluoroscopy, and in-hospital relapse after ablation. Analyzing all the above-mentioned clinical features, the CHA2DAS2VASC score summarized the impact of comorbidity as a marker of a higher recurrence rate. In this manuscript, a CHA_2_DAS_2_VASC score ≥ 3 indicated a 2.4-fold increase in recurrence.

In contrast with our study’s findings, another study^28^ indicated that the two-year recurrence-free rate was significantly better in their FPI group than in the non-FPI group. In both the success and recurrence groups of the present study, we observed a high rate of FPI, and the recurrence of these patients could be associated with non-PV triggers.

Meanwhile, another research team^29^ found that abrupt termination of RF because of esophageal temperature alerts affected acute and chronic PV reconnection. They also found that veins that caused temperature alerts and interrupted RF energy applications were no more likely to reconnect acutely or chronically than veins that had full-dose RF lesions without causing alerts. In a subsequent randomized study, the same group^30^ found that in LSI-guided CA, the use of higher power did not result in a greater number of esophageal temperature alerts or peaks than lower power. Indeed, it might have led to a lower number. High power seemed to be associated with better results from acute procedures; additionally, the lack of a higher number of esophageal alerts with the use of higher power might be associated with the delivery of better RF lesions.

A study by Yu et al.^31^ revealed that sinus rhythm HR modification was indicative of high post-AF ablation sinus rhythm maintenance with significant vagal modulation and without adverse cardiac effects. In these post-AF ablation cases, a high sinus HR was associated with a significantly lower clinical recurrence of AF after CA. These findings were corroborated by von Olshausen et al.,^32^ that showed a significant increase by 11.5 bpm immediately after ablation. Three months after ablation, the mean HR decreased slightly; however, it remained markedly higher than the pre-ablation value. They suggested that an increase in HR sinus rhythm was related to a decreased probability of recurrence. Corroborating the findings of the actual manuscript, our previous study^33^using low-power long-duration (LPLD) ablation observed recurrence in 40.4%, while in the HPSD group, recurrence was observed in only 16.5% of the patients across 30 months of follow-up. Comparing the LPLD and HPSD techniques, the latter produced a higher HR increase, with a major impact on freedom from recurrence. When we pooled together the patients that achieved higher HRs after ablation independently of the technique used, the HR increase also showed an important impact on freedom from recurrence. Another important issue addressed in the present study was the documentation of two variables in HR modulation: the inability to achieve an increase of > 10% in HR with a 4.8-fold risk of recurrence and the presence of a ≥ 30% HR increase with a 1.2-fold higher chance of sinus rhythm maintenance during our follow-up.

From our knowledge, this is the first study to document that lower LAT was an important marker of long-term success with a 2.8-fold lower risk for recurrence. This might be a reflection of a better lesion set fulfillment since PVs were isolated in a faster fashion.

As a prognostic matter, the findings of our study could be useful in the selection of patients who will need a closer follow-up with a more rigorous monitoring protocol as well as during CA since intraprocedural features can identify higher-risk patients for AF recurrence. Regarding HR modulation, it may also be useful for indicating a potential treatment modality in patients who need repeat ablation and do not have a significant change in HR.

### Study limitations

In this prospective, single-arm and single-center pilot study, the non-continuous cardiac rhythm was assessed using ECGs and 24-hour Holter monitoring to document the recurrence of atrial tachyarrhythmia. Continuous monitoring may produce different findings, but this kind of resource was not available routinely in our institutions, and symptomatic patients received more continuous monitoring with no impact in documenting atrial arrhythmias.

Another limitation of our study was the exclusion of the patients who were not converted to sinus rhythm after electrical cardioversion. Those patients have an established type of AF with higher recurrence rates that was not analyzed in the current study, and this may be a bias selection. The major reason for the selection of patients only in sinus rhythm at the beginning of the procedure, as previously described, was the need to analyze the modification of sinus heart rate after ablation. Finally, we must emphasize that when we only included patients in sinus rhythm at the beginning of the ablation could also be another form of selection bias.

## Conclusions

In this study, using HPSD and DT, we identified two success predictors: an increase of ≥ 30% in heart rate and lower time in (< 60 min) LA. Higher-risk clinical procedure features indicating recurrence were age with ≥ 65 years old, obesity, a CHA_2_DS_2_VASC score ≥ 3, persAF, and a heart rate increase of ≤ 10% after ablation.
